# Reviewed article: Guerreiro BA, Lara LS, Gialain IO, Silva CSV, Volpato LER. Analysis of the profile of patients admitted to an orofacial cleft rehabilitation service: an observational study, Cuiabá, 2005-2023. Epidemiol Serv Saude. 2025:34;e20240926

**DOI:** 10.1590/S2237-96222025v34e20240926.b

**Published:** 2025-10-24

**Authors:** Jose Mauro de Oliveira Squarisi

**Affiliations:** 1 Universidade Federal de Uberlândia, Uberlândia, MG, Brazil

## Peer review B

## Questionnaire

Does the manuscript contain new and significant information to justify publication? Yes

Does the Abstract (Summary) clearly and accurately describe the content of the article? Yes

Is the problem significant and concisely stated? Yes

Are the methods described comprehensively? Yes

Are the interpretations and conclusions justified by the results? Yes

Is adequate reference made to other work in the field? Yes

## Manuscript Structure

Length of article is: Adequate

Number of tables is: Adequate

Number of figures is: To few

Please state any conflict(s) of interest that you have in relation to the review of this paper. None

Interest: Excellent

Quality: Excellent

Originality: Good

Overall: Excellent

Recommendation: Reject

## Comments

Algumas informações estavam com texto mais longo (por exemplo referente as características embriológicas de formação das fissuras labiopalatinas) e algumas informações talvez pudessem ser retiradas (com relação ao hospital ser referências para o estado, por exemplo), porém optei por não sugerir alterações devido ao perfil da revista avaliar os serviços de saúde e as análises epidemiológicas. Sugeriria alterações para reduzir estas informações ao mesmo artigo caso fosse direcionado a um jornal/revista específico de cirurgia plástica ou crânio-maxilo-facial. Portanto não vejo necessárias alterações da forma que o artigo foi enviado.

